# Fresh-Cut Pineapple as a New Carrier of Probiotic Lactic Acid Bacteria

**DOI:** 10.1155/2014/309183

**Published:** 2014-06-29

**Authors:** Pasquale Russo, Maria Lucia Valeria de Chiara, Anna Vernile, Maria Luisa Amodio, Mattia Pia Arena, Vittorio Capozzi, Salvatore Massa, Giuseppe Spano

**Affiliations:** ^1^Department of Agricultural, Food and Environmental Science (SAFE), University of Foggia, Via Napoli 25, 71122 Foggia, Italy; ^2^Promis Biotech s.r.l., Via Napoli 25, 71122 Foggia, Italy

## Abstract

Due to the increasing interest for healthy foods, the feasibility of using fresh-cut fruits to vehicle probiotic microorganisms is arising scientific interest. With this aim, the survival of probiotic lactic acid bacteria, belonging to *Lactobacillus plantarum* and *Lactobacillus fermentum* species, was monitored on artificially inoculated pineapple pieces throughout storage. The main nutritional, physicochemical, and sensorial parameters of minimally processed pineapples were monitored. Finally, probiotic *Lactobacillus* were further investigated for their antagonistic effect against *Listeria monocytogenes* and *Escherichia coli* O157:H7 on pineapple plugs. Our results show that at eight days of storage, the concentration of *L. plantarum* and *L. fermentum* on pineapples pieces ranged between 7.3 and 6.3 log cfu g^−1^, respectively, without affecting the final quality of the fresh-cut pineapple. The antagonistic assays indicated that *L. plantarum* was able to inhibit the growth of both pathogens, while *L. fermentum* was effective only against *L. monocytogenes*. This study suggests that both *L. plantarum* and *L. fermentum* could be successfully applied during processing of fresh-cut pineapples, contributing at the same time to inducing a protective effect against relevant foodborne pathogens.

## 1. Introduction

A challenge for the food industry over the coming years is trying to meet the increasing demand for foods that encompass several levels of quality attributes including safety, nutritional, and health value. Fresh-cut fruits and vegetables respond well to these requirements and their acceptance tends to be higher among specific categories of consumers [1]. In recent years, some attempts were made to further improve the added value of minimally processed fruits and vegetables, proposing them as functional foods. Thus, juices matrices have been proposed as carrier for probiotic microorganisms [2, 3], and few examples of minimally processed fruits such as papaya and apple slices were enriched with commercial probiotic bacteria [4–6]. In fresh-cut fruit processing, typical operations such as peeling and cutting can promote microbial adhesion to the tissue, increasing the surface contact and the release of cellular content rich in minerals, sugars, vitamins, and other nutrients, ideal substrates for probiotic bacteria growth [7]. Fresh fruits and vegetables contain mostly cellulose, which is not digested by humans and may play a protective role for probiotic microorganisms in the gastrointestinal system [6, 8, 9]. Standing to these considerations, an increasing interest for fresh-cut fruits as potential matrices to vehicle beneficial microorganisms is arising, which can be also considered a promising alternative to probiotic dairy products [10].

From a microbiological point of view, it is known that minimally processed fruit and vegetables can be a risk for the safety of the consumers [11]. Foodborne illnesses are mainly related to the consumption of fresh-cut products contaminated by* Listeria monocytogenes* and* Escherichia coli* O157:H7 [12, 13]. Although the high acidity would hinder the proliferation of pathogens on fresh-cut products, growth of* E. coli* O157:H7 and* L. monocytogenes* was reported on several minimally processed fruits such as apples [14–16], peaches [17], mangoes [18], oranges [19], and strawberries [20]. Nonacidic fruits as melon, watermelon, papaya, and persimmon have also shown to be a good substrate for foodborne pathogens' growth [18, 21, 22].

For this reason, several methods have been developed in the last years to fight the growth of pathogenic microorganisms including both chemicals and biological approaches [23]. Among the green strategies, the employment of antagonistic bacteria, particularly lactic acid bacteria (LAB), as biocontrol agents against human pathogens on fresh produce has been reported with encouraging results [24].

In this work, we proposed fresh-cut pineapple as a new carrier to drive potential probiotic strains belonging to* L. plantarum* and* L. fermentum* species. The main nutritional, physicochemical, and sensorial features of pineapple pieces were also monitored to determine if the probiotic LAB used in this study would affect the overall quality of the fresh-cut product throughout storage. The same microorganisms were also investigated for their antagonistic effect against* L. monocytogenes* and* E. coli* O157:H7.

## 2. Materials and Methods

### 2.1. Bacterial Strains and Growth Conditions

*Lactobacillus plantarum* B2 (CECT 8328) and* Lactobacillus fermentum* PBCC11.5 (CECT 8448), previously isolated from sourdoughs [9, 25] and deposited at the Spanish Type Culture Collection (Valencia, Spain), were routinely grown on MRS broth (Oxoid, Hampshire, UK) at 30°C.

The type strains* Listeria monocytogenes* CECT 4031 and* Escherichia coli* O157:H7 CECT 4267 used for the antagonistic assays were grown on TSB at 37°C.

### 2.2. Preparation of the Probiotic Solution

The probiotic solution was obtained as reported by Rößle et al. [5]. Briefly, microbial strains were inoculated from a cryopreserved stock (1 : 1000 v/v) in 4 L of MRS broth and incubated at 30°C until the late-exponential phase (OD_600_ = 3.5) corresponding to approximately 8 × 10^9^ CFU mL^−1^ according to previously generated standard curve. Then, cells were recovered by centrifugation (5,000 rpm × 5 min), washed twice with citric acid-sodium citrate buffer (pH 3.8) (Sigma-Aldrich, St. Louis, MO, USA), and resuspended in 2 L of the same buffer to obtain a final concentration of 1 × 10^10^ CFU mL^−1^. Inoculum concentration was checked by plating appropriate dilutions onto MRS agar.

### 2.3. Inoculation of Pineapple Pieces with Probiotics Bacteria

Pineapple fruits (*Ananas comosus* L.), purchased at local markets (Foggia, Italy), were stored at 12°C until the assays. Fruits were sorted to eliminate damaged or defective samples and washed in tap water. Peel was manually removed with a ceramic knife, then the fruits were cored and the pulp was cut into 1 cm thick wedges. From each wedge, 8 pieces were obtained. Forty-five pieces randomly selected for each treatment were dipped for 2 min in agitation in approximately 700 mL of buffer solution (citric acid-sodium buffer, pH 3.8) containing* L. plantarum* or* L. fermentum*, respectively. Control samples were plunged only in the buffer solution. After treatment, pineapple pieces were air-dried, packed in polypropylene plastic film bags (10 × 10 cm, OTR of 1100 cm^3 ^m^2^ 24 h^−1^ bar^−1^) each containing 15 pineapple pieces, and thermally sealed in passive-modified atmosphere packaging. Analysis was performed after 0, 3, 6, and 8 days of storage at 5°C. All treatments were performed in triplicate.

### 2.4. Determination of the Microbial Load in Artificially Contaminated Pineapple Pieces

For microbiological enumeration, three pieces of each treatment were weighted, diluted (1 : 10) with saline solution (NaCl 8.6 g L^−1^), and homogenized in a blender (Bag Mixer, Interscience, Saint-Nom-la-Bretèche, France) for 2 minutes. Then, samples were submitted to tenfold serial dilution.* L. plantarum* and* L. fermentum* concentration was determined by plating on MRS agar after incubation at 30°C for 48 h. Mesophilic microorganism and yeasts and moulds were enumerated by plate counting on PCA or PDA (Oxoid) added with chloramphenicol (100 mg L^−1^) and incubated at 25 and 30°C for 48 h, respectively.

### 2.5. Antagonistic Assays

Pineapples wedges were made as previously reported. From each wedge, plugs (1.5 cm × 1.5 cm) were obtained with a corel. Samples were stored at 5°C until analysis.

Microorganisms at middle exponential phase (OD_600_ = 0.8) were collected after centrifugation (5,000 rpm × 5 min), washed twice, and then resuspended in 10 mL of sterile saline solution. Viability of the microbial solution was checked by plate counting analysis.

Each pineapple plug was spread with 15 *μ*L of solution containing 2 × 10^7^ and 2 × 10^8^ CFU mL^−1^ of pathogenic and probiotic bacteria, respectively. Controls were represented by pineapples inoculated with the same concentration of single microorganism. the microbial load was monitored at 0, 2, 5, and 7 days on pineapples plugs stored at 5°C. MRS plate agar was used to count* L. plantarum* and* L. fermentum* after appropriate serial dilutions.* E. coli* O157:H7 and* L. monocytogenes* were enumerated on TSA supplemented with 200 *μ*g mL^−1^ ampicillin or Palcam Agar, respectively.

### 2.6. Physicochemical Analysis

#### 2.6.1. Color Analysis

Color was measured using a spectral scanner (DV, Padova, Italia). The external surfaces of ten pineapple pieces for each replicate were scanned. The central region was manually selected. On these regions, color in CIE L*a*b* scale was measured. From the primary L*, a*, and b* values, the following indexes were calculated.

Hue angle:

(1) $$\begin{array}{c} h^{{}^{\circ}}=\arctan\frac{b^{*}}{a^{*}}. \end{array}$$

Global color variation:

(2) $$\begin{array}{c} \Delta E=\sqrt{{\left(L_{0}^{*}-L_{t}^{*}\right)}^{2}+{\left(a_{0}^{*}-a_{t}^{*}\right)}^{2}+{\left(b_{0}^{*}-b_{t}^{*}\right)}^{2}}. \end{array}$$

#### 2.6.2. Gas Composition

Oxygen and carbon dioxide percentage inside the bags was measured in the headspace of each sample replicate using a handheld gas analyser (CheckPoint, Dansensor A/S, Denmark) during the storage time.

#### 2.6.3. Firmness

Ten pieces for each replicate were cut into small cubes (10 mm side length) and compressed between two parallel plates using an Instron Universal Testing Machine (model 3340), with a crosshead speed of 30 mm min^−1^. Firmness of the fruit samples was defined as the rupture load of the force/deformation curve and expressed in Newton (N).

#### 2.6.4. Total Phenols and Antioxidant Capacity

Fruit extracts were obtained by homogenizing 15 g of pineapples in an Ultraturrax (IKA, T18 Basic; Wilmington, NC, USA) for 1 min with 20 mL of extraction medium, 2 mM NaF methanol : water solution (80 : 20). The homogenate was filtered through 2 layers of cheesecloth and then centrifuged at 5°C at 9,000 rpm for 5 min. The supernatant was used to analyse total phenols and antioxidant activity. Total phenols were determined according to the method of Singleton and Rossi [26]. The content of total phenols was expressed as milligrams of gallic acid per 100 grams of fresh weight (mg GA 100 g^−1^). Antioxidant assay was performed following the procedure described by Brand-Williams et al. [27] with minor modifications. The diluted sample, 50 *μ*L, was pipetted into 0.950 mL of DPPH solution to initiate the reaction. The absorbance was read at 515 nm after overnight incubation. Trolox was used as a standard and the antioxidant activity was reported in mg of Trolox equivalents per 100 g of fresh weight (mg TE 100 g^−1^).

#### 2.6.5. Simultaneous Analysis of Organic Acids and Sugars

Organic acids and sugars were extracted homogenizing 15 g of fresh pineapple tissue with 15 mL of ultrapure water for 1 min. The homogenate was centrifuged at 9,000 rpm for 10 minutes at 5°C. The supernatant was filtered with a C_18_ Sep-Pak cartridge (Grace Pure, New York, USA) and then with a 0.2 *μ*m filter (Incofar, Modena, Italy). Organic acids and sugars were identified using the method as described by Mena et al. [28]. The different organic acids and sugars were characterised and quantified by chromatographic comparison with analytical standards. Sugars and organic acids contents were expressed as g per 100 g or mg per 100 g of fresh weight, respectively.

#### 2.6.6. Total Soluble Solids, Titratable Acidity, and pH

Total soluble solids contents (TSS) were measured with a digital hand refractometer (Atago, Japan). For pH and titratable acidity (TA), 5 g of juice was titrated with an automatic titrator (TitroMatic 1S, Crison, Spain). TA was expressed as percent of citric acid (applying the acid milliequivalent factor 0.064 resp.) referred to the juice.

#### 2.6.7. Vitamin C

Vitamin C content was assessed homogenising 5 g of pineapple tissue for 1 min with 5 mL of methanol/water (5 : 95), plus citric acid (21 g L^−1^), EDTA (0.5 g L^−1^), and NaF (0.168 g L^−1^). The homogenate was filtered and the pH was adjusted to 2.2–2.4 by addition of 6 mol L^−1^ HCl. The homogenate was centrifuged at 10,000 rpm for 5 min and the supernatant was recovered, filtered through a C18 Sep-Pak cartridge (Waters, Milford, MA, USA) and then through a 0.2 *μ*m cellulose acetate filter. L-ascorbic acid (AA) and L-dehydroascorbic acid (DHAA) contents were determined as described by Zapata and Dufour [29] with some modifications [30]. AA and DHAA contents were expressed as mg of L-ascorbic or L-dehydroascorbic acid per 100 g of fresh weight.

#### 2.6.8. Sensorial Quality

A panel of six trained panelists carried out the sensory evaluations of fresh-cut pineapple at the processing day and at each sampling time. Translucency, dehydration, browning, flavour, firmness, juiciness, sweetness, acidity, off-flavour, off-odors, and color were evaluated using an hedonic scale from 1 to 5, where 1 = not present/very low/not typical and 5 = very pronounced/very typical of fresh fruits. For overall appearance, a photographic scale was used, which included 1 picture and a brief description for each point, with 1 =* really poor*; 2 =* browned flesh and translucent areas (limit of edibility)*; 3 =* yellow flesh, slightly translucent areas (limit of marketability)*; 4 =* bright yellow flesh*; 5 =* excellent*. Every attribute was scored on a 1 to 5 scale, where 1 =* absent*, 3 =* moderate*, and 5 =* full characteristic or fresh*.

### 2.7. Statistical Analysis

The effect on quality parameters of treatment was tested by performing a one-way ANOVA using StatGraphics Centurion XVI.I (StatPoint Technologies, Inc., USA), and mean values within each sampling were separated applying Tukey's test with significant difference when *P* ≤ 0.05. Analysis of variance was performed separately for each sampling day.

## 3. Results

### 3.1. Survival of Probiotic Strains in Fresh-Cut Pineapple

**Figure 1:**
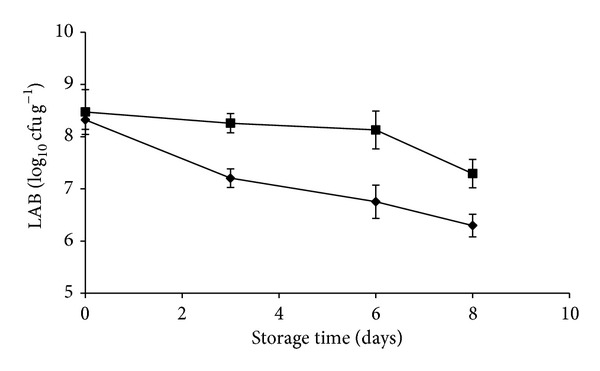
Population of* L. plantarum* B2 (square) and* L. fermentum* PBCC11.5 (diamond) on artificially inoculated pineapples stored at 5°C for 8 days. Experiments were performed in triplicate, and the standard deviations are indicated.

*L. plantarum* B2 and* L. fermentum* PBCC11.5 were tested for their ability to survive in pineapple pieces at refrigeration temperature during 8 days of storage. Strains were independently inoculated at a concentration of about 8.4 ± 0.42 log 10 cfu g^−1^. A reduction in the survival of both inoculated strains was always observed. However, while* L. fermentum *achieved a final level of 6.3 ± 0.22 log 10 cfu g^−1^; the survival of* L. plantarum* was higher (Figure 1). Plate count on MRS of uninoculated pineapple pieces revealed an initial contamination of about 3.5 ± 0.37 log 10 cfu g^−1^ (data not shown).

**Figure 2:**
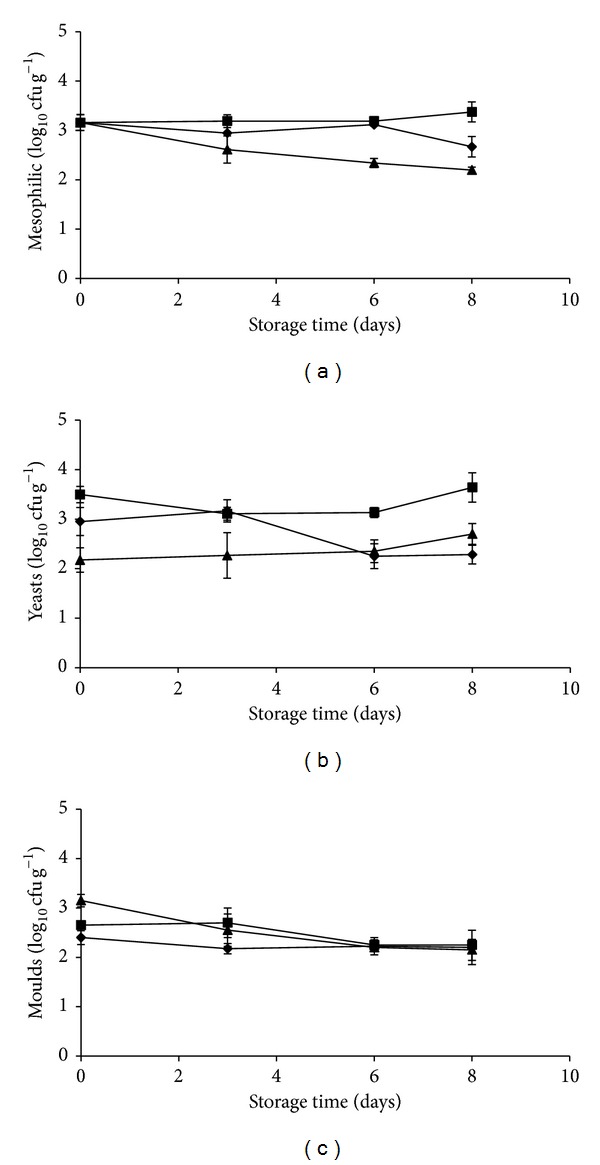
Population of mesophilic microorganisms (a), yeasts (b), and moulds (c) on fresh-cut pineapples untreated (diamond) or inoculated with* L. plantarum* B2 (square),* L. fermentum* PBCC11.5 (triangle) and stored at 5°C for 8 days. Assays were performed in triplicate, and the standard deviations are indicated.

Initial mesophilic population of uninoculated pineapple pieces was 3.5 ± 0.16 log 10 cfu g^−1^. This concentration remained almost stable during the 8 days of storage in control samples and when* L. plantarum *was added. In contrast, a reduction of about 1 log was observed in samples inoculated with* L. fermentum *(Figure 2(a)). Similarly, yeast and moulds were found at an initial contamination level of 3.68 ± 0.42 log 10 cfu g^−1^ and no differences were found in their growth either during the storage time or in the presence of probiotic bacteria (Figures 2(b) and 2(c)).

### 3.2. Quality Evaluation

At the time of processing, pineapples had solid soluble content equal to 12%, juice pH of 3.52, and titratable acidity of 0.68%, expressed as citric acid.

**Figure 3:**
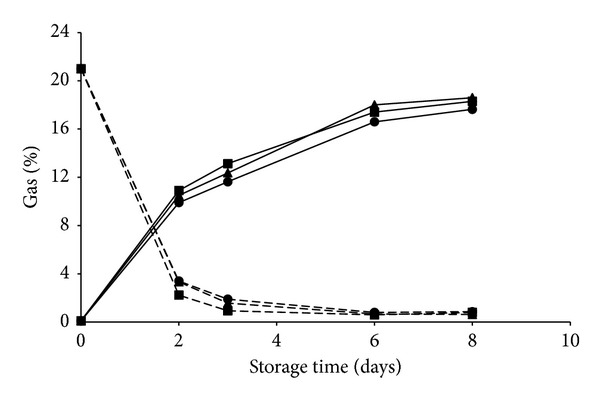
In-package atmosphere changes of O_2_ (dashed lines) and CO_2_ (continuous lines) of fresh-cut pineapples untreated (circle) or inoculated with* L. plantarum* B2 (square),* L. fermentum* PBCC11.5 (triangle) and stored at 5°C for 8 days. Data are means of three replicates for each sampling time.

Regarding the gas evolution inside the bags, slight differences were found between the treated and control samples. Oxygen concentration dropped after two days of storage and then remained quite stable around values of 0.6–0.8% up to the end of storage time. Carbon dioxide reached maximum values of about 18% in all the bags with a slightly higher, but not significant, increase in samples inoculated with* L. plantarum* and* L. fermentum* (Figure 3).

Probiotic bacteria had a minimal effect on quality and composition of pineapple fruit pieces; however, some differences were observed in terms of color and overall appearance.

**Figure 4:**
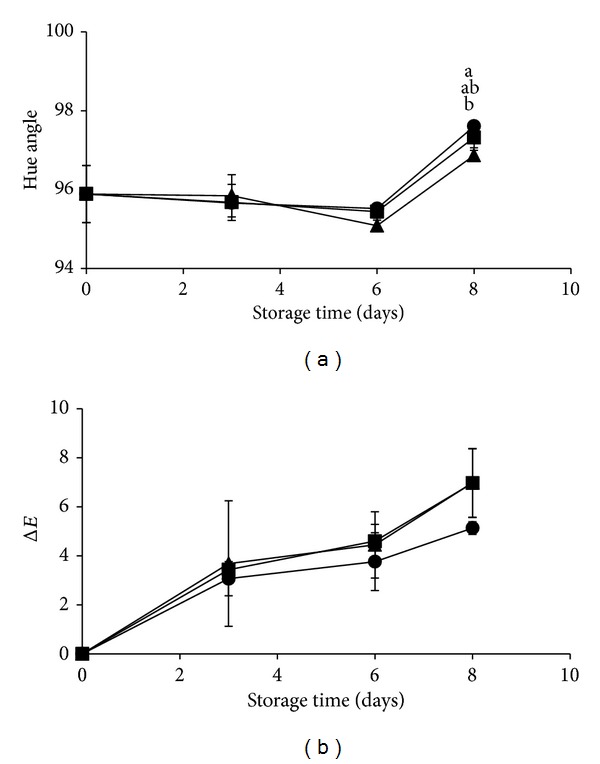
Color parameters evolution (Hue angle (a), Δ*E* (b)) of fresh-cut pineapple pieces untreated (circle) or inoculated with* L. plantarum *B2 (square),* L. fermentum *PBCC11.5 (triangle), and stored for 8 days at 5°C. Reported values are means of ten pieces for each replicate for each sampling time. Means with different letters at the same time of storage are significantly different according to Tukey's test (*P* value ≤0.05).

Color of the pieces showed significant differences in terms of a* and L* values (data not shown) and consequently on Hue angle and Δ*E* variations. Particularly, pineapple pieces inoculated with* L. fermentum* showed at the end of the storage less variation of a* values than control samples (data not shown), which in turn induced less color variation. Hue angle increased during storage for all treatment going from 96 to 98°C, meaning a reduction of the yellow component, but with a minor extent for pieces inoculated with* L. fermentum *than for control pieces, while pieces inoculated with* L. plantarum* showed intermediated results (Figure 4(a)). This difference was not evident in terms of global color variation expressed as Δ*E* (Figure 4(b)), where the change in L* values is accounting for the major part of the variation. The samples treated with the probiotic strains, in fact, showed a higher reduction of L* values (data not shown) and, as a consequence, higher value of Δ*E* compared to the untreated pineapples.

**Figure 5:**
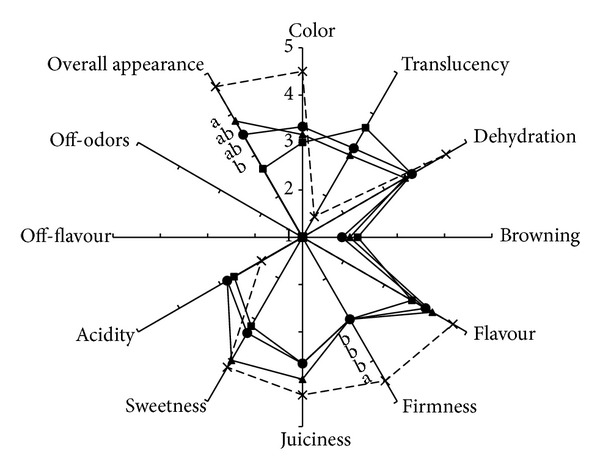
Sensory properties of fresh-cut pineapple pieces inoculated with* L. plantarum* B2 (square),* L. fermentum* PBCC11.5 (triangle), or not inoculated (circle) and stored for 8 days at 5°C. Dashed line is referred to the initial values (day 0). Reported values are means of three replicates for each sampling time and they are expressed by using an hedonic scale from 1 to 5 (1 = not present/very low/not typical and 5 = very pronounced/very typical). Means with different letters are significantly different according to Tukey's test (*P* value ≤0.05).

From a sensorial point of view, dipping in probiotic-enriched solution did not significantly affect either the organoleptic characteristics of fresh-cut pineapples or most of the external attributes (color, translucency, and browning), despite some difference observed instrumentally for color. As reported in the radar graph (Figure 5), the panelists did not observe any off-flavour or off-odor development in all the samples at the end of storage, as well as any sign of browning. Compared to initial values, the judges observed a significant (*P* ≤ 0.05) reduction of firmness and overall appearance after 8 days. Moreover, control and samples inoculated with* L. fermentum* still maintained a score higher than the limit of marketability (score 3), whereas* L. plantarum* inoculated pineapple pieces reached at the end of storage an average score of two. Also for pineapple firmness, the panelists observed a significant reduction after 8 days of storage, if compared to the initial values, but without differences among the treatments. Also no difference among treatments was instrumentally observed on firmness.

The evolution of antioxidant compounds and sugar and acids is reported in Tables 1(a) and 1(b), respectively. Total phenolics after an initial decrease, remained constant until the end of the trial and reached an average value of about 31 mg 100 g^−1^ of gallic acid. The treatment with* L. fermentum* had a higher level of antioxidant capacity after 6 days of cool storage, but not at the end when samples treated with* L. fermentum* showed the lowest antioxidant capacity. Concerning the sugars and organic acids content of fresh-cut pineapple, no any significant difference was observed, except for tartaric acid (Table 1(b)). For control samples and fruit pieces inoculated with* L. plantarum,* sucrose concentration decreased during storage, from values of 2.9 to 2.35 and 1.9 g 100 g^−1^, respectively, whereas fructose and glucose increased. In pineapples pieces inoculated with* L. fermentum*, sucrose content kept constant during storage at value of about 2.5 g 100 g^−1^. Regarding the organic acids, the content of tartaric acid at the third day of storage was lower in fruits inoculated with* L. fermentum *compared to* L. plantarum.* The trends of all the monitored acids were quite variable and not dependent on the type of treatment.

### 3.3. Antagonistic Assays

**Figure 6:**
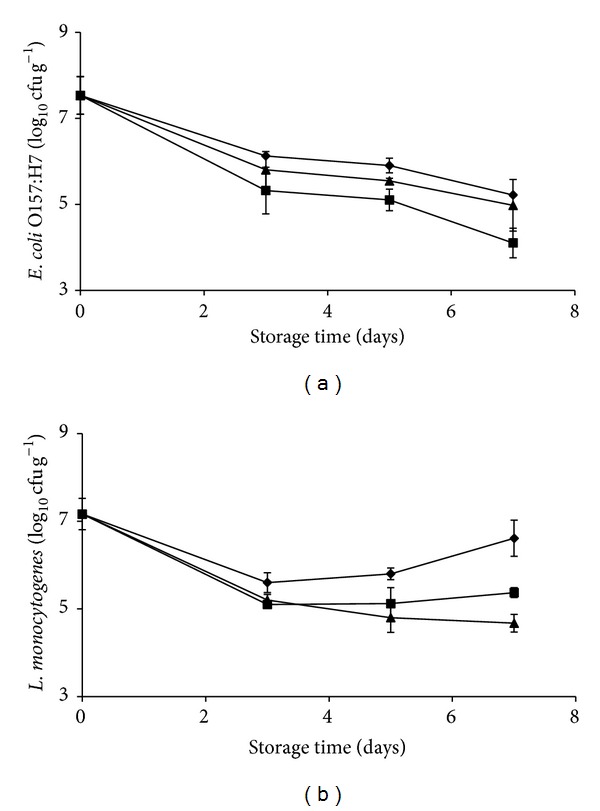
*E. coli *O157:H7 (a) and* L. monocytogenes* (b) population on pineapple pieces not inoculated (diamond) or coinoculated with* L. plantarum* B2 (square),* L. fermentum* PBCC11.5 (triangle) and stored at 5°C for 7 days. Experiments were performed in triplicate, and the standard deviations are indicated.

In order to assess the antagonistic effect of* L. plantarum* B2 and* L. fermentum* PBCC11.5 on relevant pathogenic bacteria, the growth of* L. monocytogenes* and* E. coli* O157:H7 was monitored in pineapple plugs during a time of seven days when inoculated alone or in combination with each probiotic. From an initial population of 7.53 ± 0.43 log 10 cfu g^−1^,* E. coli* CECT 4267 dropped off more than 1-log units after three days at 5°C then decreased to a final concentration of 5.21 ± 0.21 log 10 cfu g^−1^. A slight reduction of the growth was observed when* E. coli* O157:H7 was coinoculated with* L. fermentum *(4.97 ± 0.60 log 10 cfu g^−1^). Interestingly, when pineapple plugs were added with* L. plantarum*, the concentration of* E. coli* O157:H7 was lower at each experimental point and drastically reduced after 7 days of refrigeration (4.10 ± 0.14 log 10 cfu g^−1^) (Figure 6(a)).* L. monocytogenes* CECT 4031 population was 7.16 ± 0.37 log 10 cfu g^−1^ and promptly declined to about 1.5-log units and then rose at 6.61 ± 0.41 log 10 cfu g^−1^ if inoculated alone. When* L. monocytogenes* CECT 4031 was coinoculated with the antagonistic* L. fermentum* strain, a fast reduction was observed after three days, followed by a further decrease at all monitored steps until a final concentration of around 2-log units lower (4.67 ± 0.20 log 10 cfu g^−1^). In contrast, the antagonistic effect of* L. plantarum* on* L. monocytogenes* CECT 4031 was minimal at three days of storage but then increased to about 5.37 ± 0.12 log 10 cfu g^−1^ (Figure 6(b)).

**Figure 7:**
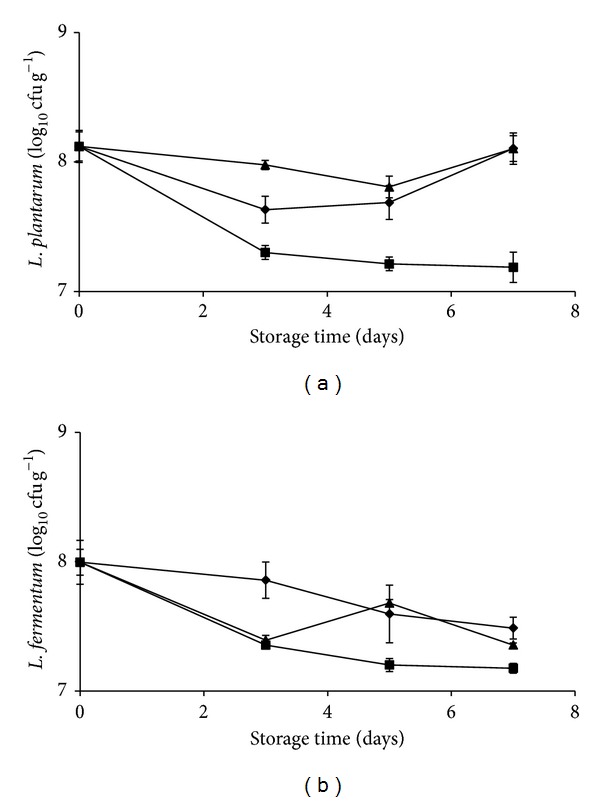
*L. plantarum* B2 (a) and* L. fermentum* PBCC11.5 (b) population on pineapple pieces inoculated alone (diamond) or coinoculated with* L. monocytogenes* (square),* E. coli* O157:H7 (triangle) and stored at 5°C for 7 days. Experiments were performed in triplicate, and the standard deviations are indicated.

*L. plantarum* population remains almost constant after 7 days of storage when inoculated alone or in combination with* E. coli* O157:H7 (Figure 7(a)). Pineapple plugs, inoculated with either* L. fermentum* or in a coinoculation approach with* E. coli* O157:H7, showed a reduction of about 0.5-log in the final level of* L. fermentum* (Figure 7(b)). In contrast, the coinoculum with* L. monocytogenes* CECT 4031 resulted in a decrease of the* L. plantarum* and* L. fermentum* microbial population of around 1-log (Figures 7(a) and 7(b)).

## 4. Discussion

Pineapple is one of the most important tropical fruits in the world, that it is often commercialized as minimally processed [31, 32]. The functionality of fresh-cut fruits could be further enhanced by proposing this kind of products as vehicle for probiotic microorganisms. To date, only few probiotic strains including the commercial* Lactobacillus rhamnosus* GG and* Bifidobacterium lactis* BB12 have been investigated to enrich fresh-cut apples and papaya [4–6]. According to this trend, for the first time, the suitability of fresh-cut pineapples as potential carrier of probiotics LAB was analyzed in the present study [9].

Probiotics can play their beneficial role if they reach the gut lumen in an enough number to provide health gain to the host, and the concentration of viable cells should be not less than 10^6^ cfu g^−1^ to be considered efficacious [33]. In accordance with this criterion, the concentration of* L. plantarum* B2 and* L. fermentum* PBCC11.5, used in this work to contaminate fresh-cut pineapples, was found sufficiently high within eight-day shelf life as already reported by other authors [4, 6, 34].

Microorganisms were inoculated on pineapples pieces by immersion in a dipping solution containing organic acid as browning inhibitor [4, 6, 34]. Dipping is a step which generally follows operations such as peeling and/or cutting to add antimicrobial, antibrowning agents, and texture preservatives [35]. Therefore, a dipping solution enriched with a high concentration of LAB could be a practical and inexpensive way to obtain minimally processed probiotic fruits.

The main physical and chemical parameters of fresh-cut pineapples were evaluated to determine if the addition of high amount of the probiotic LAB used in this study could affect the quality of the product. Beside some differences after 3 days on tartaric acid content and the antioxidant capacity, no other differences were observed on pineapple composition. Moreover, a decrease of tartaric acid, compared to control and* L. plantarum* treatments, was observed when pineapple pieces were treated with* L. fermentum*. Slight variation in color did not affect sensorial evaluation, except for overall appearance which at the end of the storage was higher in fruits inoculated with* L. fermentum* than* L. plantarum* generally which did not impact clearly the main sensorial features of minimally processed pineapples, according to results previously observed for apple wedges [5, 6]. Overall, the panelists did not observe any off-flavour or off-odor development in all the samples at the end of storage, demonstrating that high concentrations of probiotic bacteria had no effect on the degradation rate of the sensory properties of the product, and this is in accordance with the work reported by Rößle et al. [5] where panelists did not express a preference for apples containing probiotic bacteria over control apples.

Over the last years, there is clearly an urgent need to develop new and eco-friendly methods to control the postharvest increase of foodborne pathogens. Among these, biopreservation is based on the antagonistic effect of some microorganisms, including LAB, that can play a protective role in the product itself during storage [6]. Trias et al. [36] reported that eight-teen LAB strains mainly belonging to* Leuconostoc* spp. and* L. plantarum* were able to strongly inhibit the growth of foodborne human pathogens on golden delicious apples. Moreover,* L. rhamnosus* GG reduced the growth of* L. monocytogenes* of about 1-log unit on apple wedges [6]. With a similar approach, in this study, the protective effect of* L. plantarum* B2 and* L. fermentum* PBCC11.5 against* E. coli* O157:H7 and* L. monocytogenes* was analysed on fresh-cut pineapples. Recently, Alegre et al. [37] reported that* Pseudomonas graminis* CPA-7 should be coinoculated in at least the same amount of* Listeria innocua* to adequately reduce its growth. Therefore, a concentration of 10^7^ and 10^8^ cfu mL^−1^ for pathogenic and probiotic bacteria, respectively, was used to contaminate fresh-cut pineapples. The high concentration of pathogenic bacteria used in this study is consistent with that observed by Alegre et al. [38] in fresh-cut apples applying semicommercial conditions. In the aforementioned study,* L. monocytogenes* reached a concentration of approximately 7.0 log cfu g^−1^ after a week if stored at 10°C [38], supporting the importance of a correct management of the cold chain during the shelf life of fresh-cut fruits. However, cold chain abruption with extended use after expiration date is a probable scenario and an incidence between 10 and 20% of houses and commercial refrigerators working at a temperature >10°C was reported [39].

Interestingly,* L. plantarum* B2 was able to reduce the dynamic populations of both* E. coli* O157:H7 and* L. monocytogenes*, while* L. fermentum* PBCC11.5 was effective only against* L. monocytogenes*, supporting that antagonism could be a strain or species depending feature. Likewise, in a recent screening of probiotic LAB, Ramos et al. [40] observed the highest coaggregation ability with* E. coli* by a strain of* L. plantarum*, while* L. fermentum* CH58 exhibited antagonistic activity towards* L. monocytogenes*.

In conclusion, this work fits in an attempt to expand the range of both food matrices and probiotic strains in order to obtain new and more safety minimally processed foods, suggesting that probiotic LAB could be successfully employed for the elaboration of functional pineapples, contributing at the same time to carrying a protective effect against relevant foodborne pathogens.

## Figures and Tables

**Table 1a:** (a)

	Day	Antioxidant compound content				
		Ascorbic acid	Dehydroascorbic acid	Vitamin C	Total phenols	Antioxidant capacity
		(mg/100 g fw)	(mg/100 g fw)	(mg/100 g fw)	(gallic acid mg/100 g fw)	(Trolox eq. mg/100 g)
	0	20.68 ± 3.59	3.21 ± 1.25	23.89 ± 4.72	41.49 ± 5.19	42.39 ± 3.39

Control	3	16.56 ± 1.68	4.22 ± 0.54	20.78 ± 1.72	32.72 ± 5.04	50.86 ± 1.82
*L. plantarum *B2	3	12.65 ± 4.72	5.84 ± 1.53	18.49 ± 5.94	30.65 ± 4.14	51.12 ± 2.21
*L. fermentum *PBCC11.5	3	16.82 ± 3.14	5.59 ± 0.74	22.41 ± 3.16	32.26 ± 3.94	51.03 ± 3.69

Control	6	12.24 ± 2.74	4.81 ± 0.81	17.06 ± 2.47	31.42 ± 2.51	48.90 ± 1.52^b^
*L. plantarum *B2	6	11.90 ± 3.43	4.84 ± 1.31	16.74 ± 4.74	31.40 ± 4.64	48.59 ± 2.31^b^
*L. fermentum *PBCC11.5	6	11.30 ± 1.18	3.25 ± 0.29	14.55 ± 0.97	26.90 ± 2.70	55.43 ± 2.09^a^

Control	8	15.35 ± 4.52	4.99 ± 0.82	20.34 ± 4.53	30.04 ± 0.87	54.23 ± 0.10^a^
*L. plantarum *B2	8	14.48 ± 4.08	4.63 ± 0.76	19.11 ± 4.84	33.06 ± 2.21	49.80 ± 2.49^ab^
*L. fermentum *PBCC11.5	8	14.41 ± 3.77	4.04 ± 0.79	18.45 ± 4.35	30.76 ± 2.37	47.21 ± 2.77^b^

**Table 1b:** (b)

	Day	Sugars			Organic acids			
		Sucrose	Glucose	Fructose	Citric acid	Tartaric acid	Malic acid	Succinic acid
		(g/100 g fw)	(g/100 g fw)	(g/100 g fw)	(mg/100 g fw)	(mg/100 g fw)	(mg/100 g fw)	(mg/100 g fw)
	0	2.80 ± 0.24	0.53 ± 0.05	0.09 ± 0.01	149.50 ± 7.53	10.02 ± 0.47	161.50 ± 12.65	5.90 ± 2.01

Control	3	2.91 ± 0.30	0.63 ± 0.09	0.10 ± 0.01	209.20 ± 32.68	11.86 ± 1.04^ab^	165.23 ± 20.17	10.97 ± 2.39
*L. plantarum *B2	3	2.87 ± 0.36	0.65 ± 0.06	0.11 ± 0.01	197.64 ± 30.87	12.30 ± 1.04^a^	165.46 ± 10.73	11.48 ± 2.91
*L. fermentum *PBCC11.5	3	2.50 ± 0.36	0.63 ± 0.07	0.09 ± 0.01	149.64 ± 20.64	9.68 ± 0.46^b^	143.49 ± 13.05	7.02 ± 0.18

Control	6	3.19 ± 0.57	0.73 ± 0.10	0.12 ± 0.02	217.74 ± 69.87	12.17 ± 2.27	166.92 ± 29.63	11.16 ± 5.11
*L. plantarum *B2	6	2.70 ± 1.08	0.60 ± 0.16	0.10 ± 0.02	177.82 ± 29.03	11.33 ± 4.01	149.95 ± 26.03	11.01 ± 1.97
*L. fermentum *PBCC11.5	6	2.53 ± 0.38	0.72 ± 0.09	0.12 ± 0.01	190.22 ± 31.95	11.13 ± 0.47	163.92 ± 18.43	12.01 ± 2.82

Control	8	2.35 ± 0.59	0.76 ± 0.15	0.11 ± 0.03	156.63 ± 43.64	11.42 ± 4.62	158.57 ± 39.72	7.69 ± 5.09
*L. plantarum *B2	8	1.90 ± 0.04	0.65 ± 0.03	0.10 ± 0.01	175.76 ± 50.59	10.41 ± 1.52	142.42 ± 26.01	8.22 ± 4.39
*L. fermentum *PBCC11.5	8	2.48 ± 1.14	0.69 ± 0.15	0.10 ± 0.03	158.78 ± 19.06	11.60 ± 3.63	140.58 ± 32.76	11.50 ± 2.61

Reported values are means of three replicates for each sampling time. Means with different letters at the same time of storage are significantly different according to Tukey's test (*P* value ≤0.05).
